# Persistent activation of NF-kappaB related to IkappaB's degradation profiles during early chemical hepatocarcinogenesis

**DOI:** 10.1186/1477-3163-6-5

**Published:** 2007-04-19

**Authors:** Rebeca García-Román, Julio Isael Pérez-Carreón, Adriana Márquez-Quiñones, Martha Estela Salcido-Neyoy, Saúl Villa-Treviño

**Affiliations:** 1 Departamento de Biología Celular, Centro de Investigación y de Estudios Avanzados del IPN, México D.F., México

## Abstract

**Background:**

To define the NF-kappaB activation in early stages of hepatocarcinogenesis and its IkappaB's degradation profiles in comparison to sole liver regeneration.

**Methods:**

Western-blot and EMSA analyses were performed for the NF-kappaB activation. The transcriptional activity of NF-kappaB was determined by RT-PCR of the IkappaB-α mRNA. The IkappaB's degradation proteins were determined by Western-blot assay.

**Results:**

We demonstrated the persistent activation of NF-kappaB during early stages of hepatocarcinogenesis, which reached maximal level 30 min after partial hepatectomy. The DNA binding and transcriptional activity of NF-kappaB, were sustained during early steps of hepatocarcinogenesis in comparison to only partial hepatectomy, which displayed a transitory NF-kappaB activation. In early stages of hepatocarconogenesis, the IkappaB-α degradation turned out to be acute and transitory, but the low levels of IkappaB-β persisted even 15 days after partial hepatectomy. Interestingly, IkappaB-β degradation is not induced after sole partial hepatectomy.

**Conclusion:**

We propose that during liver regeneration, the transitory stimulation of the transcription factor response, assures blockade of NF-kappaB until recovery of the total mass of the liver and the persistent NF-kappaB activation in early hepatocarcinogenesis may be due to IkappaB-β and IkappaB-α degradation, mainly IkappaB-β degradation, which contributes to gene transcription related to proliferation required for neoplasic progression.

## Background

NF-kappaB/Rel family proteins participate in the transcription of several cellular genes, including oncogenes, cell cycle regulation genes, proteins related to stress, differentiation, cytokine and cytokine receptors, all of which are involved in proliferation and inflammation processes [1]. The NF-kappaB/Rel protein family includes protoncogene c-rel, p50/p105 (NF-kB 1), p52/p100 (NF-kB 2), p65 (Rel A), and Rel B. NF-kappaB which is present in the cytosol as inactive dimmer associated with the inhibitory protein IkappaB. When this last one is phosphorylated, it is broken down by the 26 S proteasome in response to a wide variety of stimuli such as tumoral necrosis factor alpha (TNF-α), phorbol myristate acetate (PMA), lipopolysaccharides (LPS), interluekine 1 (IL-1) and others. Finally, active NF-kappaB is released and translocates to the nucleus [1]. The principal isoforms of IkappaB inhibitor are IkappaB-α and IkappaB-β, even though three more proteins exist which are implicated in this process: IkappaB-γ, IkappaB-ε and Bcl-3 [2,3].

NF-kappaB has recently related to oncogenesis development because of its role in cell survival, adhesion, differentiation, apoptosis and cell growth. Many tumor and transformed cell lines, such as leukemia, lymphoma, myeloma, melanoma, prostate, colon, breast, pancreas and head and neck squamous cell carcinoma, express NF-kappaB constitutively [4]. Several varieties of tumor support the role of NF-kappaB in the oncogenesis process. Among this evidence, previous reports show the involvement of the oncogene v-rel, member of the NF-kappaB/Rel family, which when injected into young chickens, is able to induce aggressive lymphomas [5]. A process, in which NF-kappaB plays a critical role in a proliferation program, is that of hepatic regeneration. After a partial hepatectomy the remaining liver recovers the total mass of the original liver [6]. Many factors are involved in the liver regeneration program, such as the activation of post-hepatectomy factor(s) (PHF)/NF-kappaB, through which hepatocytes regulate their mitogenic program during liver regeneration [6]. This constitutive activation of NF-kappaB has been associated with the preferential degradation of inhibitor IkappaB-β. LPS and IL-1 produce persistent activation of NF-kB as a result of both IkappaB-α and IkappaB-β degradation, while other inducers, such as TNF-α and PMA cause a transient induction of NF-kappaB affecting only IkappaB-α. Indeed this constitutive NF-kappaB activation related to gradual depletion of IkappaB-β, has been shown in the human T-Cell leukemia virus type 1 Tax [7,8].

However, incomplete knowledge of the molecular mechanism involved in persistent NF-kappaB activation in cancer development has hindered the use of therapeutic targets to block the proliferation of transformed cells. On the other hand, although the activation of nuclear factors has been studied extensively in well established cancerous tissue or in transformed cell lines, the mechanisms that allow the activation of nuclear factors during the initial stages of carcinogenesis in animal models, is poorly understood.

Using a modified Resistant Hepatocyte model [9,10] to induce liver cancer, we demonstrated the persistent NF-kappaB activation and differential degradation profiles of IkappaB-α and IkappaB-β when compared to liver regeneration. By means of Western-blot, EMSA and RT-PCR analysis, we demonstrated the persistent activation of NF-kappaB in the early stages of hepatocarconogenesis, and this result correlated with the selective degradation of inhibitors IkappaB-α and IkappaB-β in contrast to the hepatic regeneration that only manifested transitory NF-kappaB activation and IkappaB-α degradation.

## Results

### Translocation induction of NF-kappaB during initial hepatocarcinogenesis

**Figure 1:**
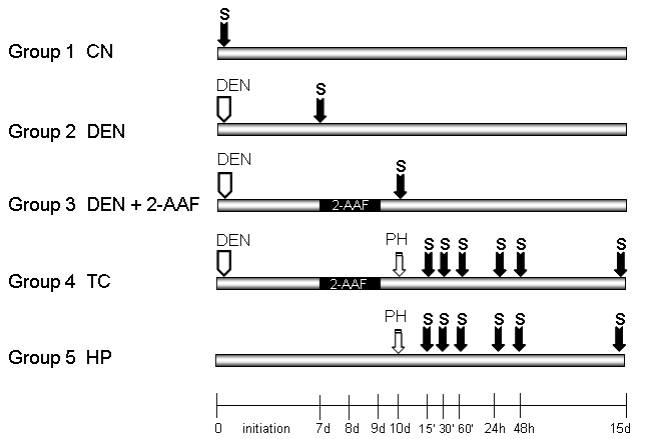
**Experimental design**. Five groups of Fisher 344 rats were subjected to different treatments according to a modified Semple-Roberts hepatocarcinogenesis model. Group 1: CN (n = 3), received no treatment. The second group (n = 3), received 200 mg/kg of DEN as initiator (ip). Group 3 (n = 3) received the same dose of DEN and on 7 d three daily serial doses of 2-AAF (20 mg/kg) orally. The fourth group (TC) (n = 18, 3 rats every time of sacrifice), received the DEN (200 mg7kg), the three daily serial doses of 2-AAF (20 mg/kg) at 7 d, before partial hepatectomy (PH) on 10 d. The fifth group received only partial hepatectomy (n = 18, 3 rats every time of sacrifice).

**Figure 2:**
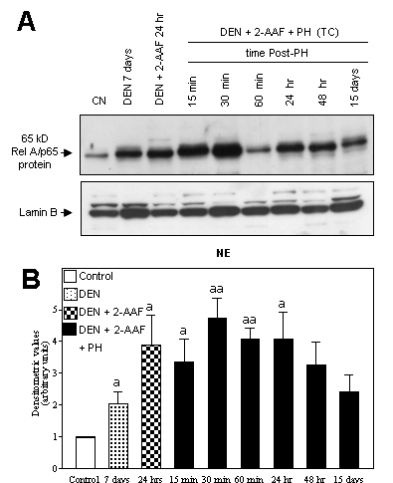
**Nuclear translocation of Rel A/p65 during early stages of hepatocarcinogenesis**. A: Western blot analysis of Rel A/p65. (CN) Normal control, (DEN) diethylnitrosamine, (2-AAF) 2-acetylaminofluorene, (PH) partial hepatectomy. B: Densitometric analysis of the 65 kD bands. All samples were normalized with lamin B. Mean values ± S.D. from three different observations. aP < 0.05, aaP < 0.005 against control, (n = 3) by ANOVA test.

We investigated early changes concerning NF-kappaB activation in the hepatocarcinogenesis model according to the scheme in figure 1. We tested the Rel A/p65 nuclear levels, present in different extracts taken from untreated animals (CN) and from DEN treatment animals, 7 d after its administration; from animals treated with DEN plus 2-AAF, sacrificed 24 h after the last dose of 2-AAF; from animals treated with DEN plus 2-AAF and PH (TC), and finally from animals with only PH. The last two groups were examined at 15, 30 and 60 min; 24 and 48 h, and 15 d after PH. Twenty μg of nuclear protein were separated on 8% SDS-polyacrilamide gel, and inmunoblot analysis was performed using pAb anti-p65, and normalized using pAb anti-lamin B (Upstate, NY and Santa Cruz Biotechnology Inc., respectively). Densitometric analysis of the 65 kD and lamin B bands in all groups was performed. Mean ± SD values from three different samples in duplicated observations was performed, where the aP values < 0.05 were considered significant. As shown in figure 2, we observed an increase in the Rel A/p65 nuclear levels from 7 d after DEN treatment with respect to normal control (CN). The DEN treatment plus the administration of three 2-AAF doses, sacrificed 24 h after the last 2-AAF dose, further incremented the Rel A/p65 nuclear levels. After complete treatment (TC), the nuclear level of Rel A/p65 increased further and reached its maximum level at 30 min after PH. Interestingly, the nuclear level of Rel A/p65 was sustained above control levels at 24 h, and even 15 d after PH.

### Persistent union of Rel A/p65 to DNA in early HC

**Figure 3:**
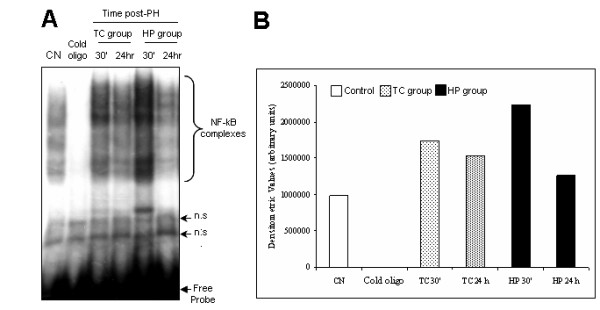
**DNA binding activity of Nuclear Factor-kappaB in hepatocarcinogenesis and liver regeneration**. A: Representative EMSA assay. B: Densitometric analysis of the NF-kappaB complexes bands in control, hepatocarcinogenesis and liver regeneration. A mixture of three different rats from each group was evaluated in a single experiment. Non specific signal (n.s.).

NF-kappaB binding activity to consensus kappaB site was analyzed by their DNA union in the EMSA assay. The nuclear extracts (10 μg) were incubated with {γ-^32^P} end-labelled consensus kB oligonucleotide using T_4 _polynucleotide kinase, followed by electrophoresis and analysis by autoradiography. For the competition assay, the cold kappaB site oligo-nucleotide was used. Samples of TC and HP groups were examined at 30 min and 24 h after PH (Figure 3A). The maximal binding activity of NF-kappaB complexes to DNA in the TC group was found 30 min after PH in 76% above the control levels (Figure 3B), this results are in agree with previous reports that shown a similar NF-kappaB complexes activation behavior [6]. There were differences in the amount of the Rel A/p65 binding activity after carcinogenic treatment, when compared with only the hepatectomyzed group. The HP group showed the same trend of maximal binding activity at 30 min, reaching 126% above control levels; however, the TC group maintained the DNA binding activity of 28% above the levels found in the HP group at 24 h after PH, indicating a persistent activation of NF-kappaB complexes during the initial steps of hepatocarcinogenesis in contrast to hepatic regeneration.

### IkappaB-α expression is sustained in early steps of hepatocarcinogenesis

**Figure 4:**
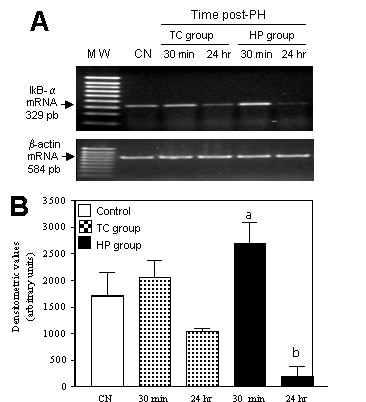
**Expression of IkappaB-α mRNA in hepatocarconogenesis and liver regeneration**. Expression levels of IkappaB-α mRNA in early stages of hepatocarcinogenesis and during liver regeneration by semiquantitative RT-PCR. A: Representative agarose gels. B: Densitometric analysis of the IkappaB-α RNAm bands was quantified and normalized according to α-actin mRNA and expressed as mean ± S.D. (n = 3) aP < 0.05 against control, bP < 0.05 compared 24 hr post-HPx in TC against HP group by Student's t test.

After detecting the persistent binding activity of nuclear NF-kappaB in the initial steps of hepatocarcinogenesis, we searched for an indication of transcriptional activity carried out by NF-kappaB. Studies indicated that *I kappa B alpha *gene is exclusively transcribed by the 65-kilodalton transactivating subunit of NF-kappaB [11,12], then we compared the levels of IkappaB-α mRNA by semiquantitative RT-PCR of TC group in contrast to the HP group (Figure 4A). Total RNA (1 μg) was performed; the amplification products of the 329 pb, were determined by means of electrophoresis in 2% agarose gel stained with ethidium bromide. The densitometric analysis of the IkappaB-α RNAm bands was quantified and normalized according to α-actin mRNA and expressed as mean ± SD aP < 0.05 against control, bP < 0.05 compared 24 h post-PH in TC against HP group by Student's t test. We observed an 18% increase in IkappaB-α mRNA levels 30 min after PH in the TC group, (Figure 4B), however, it was not significant. The sole PH in the HP group after 30 min produced a higher IkappaB-α expression level of 55% above levels of non-treated rats. Nevertheless in the TC group, after 24 h the mRNA levels diminished to a level only 40% below that of non-treated rats, while after the sole PH, the transcriptional levels dramatically decreased to 88.47%, when comparing both groups to that of non-treated rats (Figure 4B). These latter differences are statistically significant and strongly suggest that transcriptional activity of NF-kappaB in early steps of hepatocarcinogenesis is increased and sustained at 24 h in contrast to that observed after PH which showed only a transitory increase.

### Degradation of IkappaB-α and IkappaB-β in early HC whereas hepatic regeneration affects only IkappaB-α

**Figure 5:**
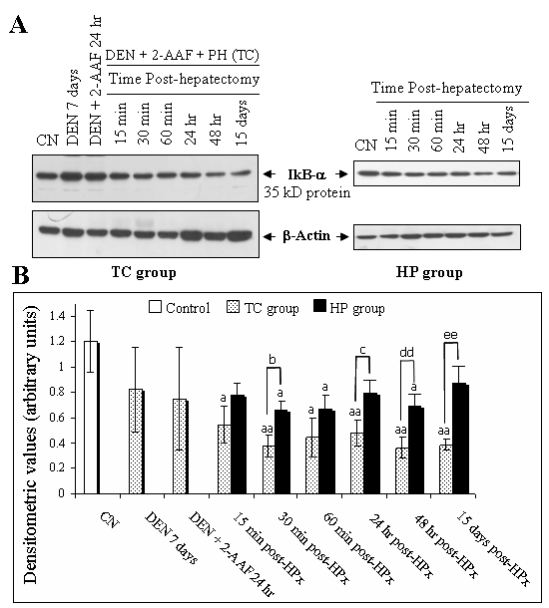
**Hepatocarcinogenesis treatment causes acute and sustained degradation of IkappaB-α**. A: Representative Western blotting of the cytosolic degradation of IkappaB-α during early stages of the hepatocarcinogenesis model. B: Densitometric analysis of the 35 kD bands. Mean values ± S.D. of three different observations. aP < 0.05, aaP < 0.005 against control, bP = 0.05, bbP = 0.005 against respectively bar, (n = 3) by ANOVA test.

**Figure 6:**
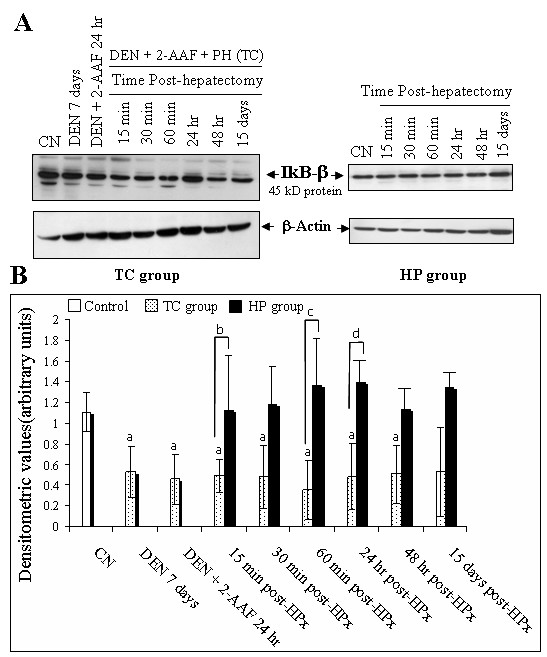
**The liver regeneration not induces the IkappaB-β degradation while the hepatocarcinogenesis treatment causes a persistent IkappaB-β degradation**. A: Representative Western blotting of the cytosolic degradation of IkappaB-β. B: Densitometric analysis of the 45 kD bands. Mean values ± S.D. of three different observations. aP < 0.05, aaP < 0.005 against control, bP = 0.05, bbP = 0.005 against respectively bar, (n = 3) by ANOVA test.

Previous studies have indicated that activation of NF-kappaB correlates with IkappaB's degradation [13]. Therefore, we assessed protein levels of IkappaB-α and IkappaB-β from liver cytosolic extracts in the initial stages of hepatocarcinogenesis in comparison with liver regeneration. The IkappaB cytosolic protein level was assayed by means of Western blotting. Twenty μg of rat liver cytosolic extracts were separated on 12% SDS-PAGE, and inmunoblot analysis was performed using the IkappaB-α and IkappaB-β antibodies. Densitometric analysis of the 35 and 45 kD bands respectively in all groups was performed. The protein level of IkappaB-α of the TC group 7 d after DEN administration or at 10 d, 24 h after the last dose with 2-AAF, revealed that IkappaB-α levels were not altered (Figure 5). This situation may be a consequence of a rapid recovery of the protein levels. On the other hand, on the same day 10, the IkappaB-α protein was rapidly degraded 15 min after PH, and reached the maximal degradation level at 30 min post-PH. Interestingly, the decrease of the IkappaB-α protein continued for up to 24 and 48 h and its levels were below those of non-treated rats, even 15 d after PH. Similarly, the 45 kD IkB-β band in the protein of the TC group was affected but later (60 min) after PH, and its levels remained low even at 24 and 48 h (Figure 6). This experiment highlighted differences in the degradation kinetics between IkappaB-α and IkappaB-β. According to our results, a similar behavior has been shown in relation to the NF-kappaB activity, which reaches its maximum within 30 min and decays rapidly within 4 h to control levels in 70Z/3 cells stimulated by TNF-α [14]. We observed that the NF-kappaB activity persisted for over 15 d post-PH in the initial phases of hepatocarcinogenesis, and IkappaB-α and IkappaB-β decrease displayed similar behavior. Once the degradation profile of both IkappaB-α and β was observed in initial steps of hepatocarcinogenesis, we determined their degradation profile at 15, 30 and 60 min, 24 and 48 h and 15 d after only PH. The PH treatment caused a loss of IkappaB-α protein which reached its maximal peak at 30 min post-PH, and was sustained 24 h after surgery (Figure 5). Degradation of IkappaB-α was higher in the TC group in comparison to the liver regeneration group. There were clear differences between both groups, at 30 min, 24 and 48 h post-PH. Intriguingly, there was no effect on IkappaB-β degradation in the post-PH profile at any time analyzed (Figure 6). These results suggest the existence of a different mechanism regulating NF-kappaB activation in the initial stages of chemical hepatocarcinogenesis, as opposed to liver regeneration, and as reflected by differential degradation of IkappaB's.

## Discussion

The fact that interventions during the first 10 d in the modified Resistant Hepatocyte Model (RH) [10], which includes a partial hepatectomy as proliferative stimulus, are sufficient for inducing liver cancer permit us to study of the molecular pathways during the progress of neoplastic foci towards malignant cancer [9]. We hereby report NF-kappaB activation and the degradation profiles of IkappaB during these initial 10 d of chemical hepatocarcinogenesis. In this period we find a persistent NF-kappaB activation response, as compared to activation observed after PH alone. Our results agree with previous reports that indicate that NF-kappaB activation persists 25 d and even 28 d after initiating treatment with modified RH model, and remains activated even during more advanced stages or in cancerous liver tissue [10,15]. Some studies indicate that NF-kappaB is activated by several carcinogens and tumor promoters that include benzo(a)pyrene, UV radiation and phorbol esthers (PMA) [16,17]. Moreover, tumor cells have been found to express a continuous NF-kappaB activation. This has been shown in cells ranging from leukemia, lymphoma, myeloma, and prostate and breast cancer [5]. We hypothesized a similar persistent NF-kappaB activation in the modified RH model during early stages of hepatocarcinogenesis, and therefore decided to explore NF-kappaB activation after DEN treatment, during promoter administration and further on after PH. The Rel A/p65 nuclear levels in nucleus was incremented seven days after DEN administration, and the maximal level was found at 10 d, 30 min after PH. Previous reports shown a high increment of NF-kappaB activation by 30 min post-PH, but decayed drastically after 60 min [6]. However, our results in the TC group showed that the level of Rel A/p65 displayed a maximal translocation at 30 min after PH, which was sustained above the basal levels even after 15 d. It is noteworthy in the TC group that NF-kappaB/DNA association and the mRNA levels of IkappaB-α increased at 30 min, and this transcriptional activity was sustained above the level in hepatectomy after 24 h. After the sole PH the IkappaB-α mRNA levels diminished beyond basal levels at 24 h, possibly due to auto-regulatory feedback which blocks the NF-kappaB activation, through a temporal increment of IkappaB-α synthesis.

IkappaB-α and IkappaB-β respond differently to inducers of NF-kappaB activity. For instance TNF-α and PMA produce rapid and transient activation of NF-kappaB which only affects IkappaB-α complexes, whereas LPS and IL-1 cause persistent activation of NF-kappaB affecting both, IkappaB-α and β complexes [7]. It seems that in our carcinogenic model involving a PH as proliferative stimulus, NF-kappaB responds differently as compared with a sole PH. Previous stages in the PH in the modified hepatocarcinogenesis model did not cause a diminution of the protein levels of IkappaB-α; we speculate that this response was due to the new re-synthesis of IkappaB-α. However, the profile of IkappaB-α levels was different after PH; in the TC group it began to decrease at 15 min, the lowest concentration was reached at 30 min, at it was maintained even after a further 15 d. In the liver regeneration, we showed an IkappaB-α decrease of cytosolic protein levels with a minimum at 30 min as reported elsewhere [6], whereas, the magnitude of this decrement was greater in the TC group in comparison to liver regeneration. The most striking difference after PH in the TC group in comparison to that of liver regeneration, was the preferential degradation of IkappaB-β in early HC, low levels of IkappaB-β appeared from 7 d after DEN administration, remained low at 24 h after the last dose with 2-AAF, in rats treated with DEN plus 3 doses of 2-AAF, and reached its lowest concentration 60 min after PH. In comparison, in liver regeneration there were no changes in cytosolic protein levels at any of the time analyzed. From the literature it is quite clear that IkappaB-β, has a slower degradation than IkappaB-α, which agrees with our results. Previous reports have shown that IL-1 induced a slower IkappaB-β degradation than IkappaB-α in glial cells, accompanied with a persistent long term activation of NF-kappaB [18]. Furthermore, IkappaB-β has been shown to be a less-efficient substrate for the IkappaB-kinase (IKK) when compared with IkappaB-α, therefore, it has been proposed that the IkappaB-β has a slower rate of phosphorylation kinetic [19]. It has been suggested that IkappaB-β phosphorylation may be a critical stage in the regulation of inhibition activity [20,21]. In a hyperphosphorylated state, IkappaB-β is degrades by means of prior ubiquitination, but the inhibitory activity is displayed [22,23] in a phosphorylated form and in the case of hypophosphorylate, the domain of nuclear localization of Rel A/p65 is not masked, permitting NF-kappaB/IkappaB-β complexes to enter the nucleus and support the persistent NF-kappaB activation [24].

Another point of consideration in our study was the IkappaB-α mRNA diminution at 24 h after PH, which is less pronounced in the hepatocarcinogenesis group in contrast to the group which was only hepatectomized. It has been suggested that upon activation of NF-kappaB, the newly synthesized IkappaB-α attenuates the NF-kappaB nuclear translocation, entering the nucleus and displacing NF-kappaB bound to DNA [11]. In an attempt to explain this phenomenon, we hypothesized the coexistence of inactive binary complexes IkappaB-α/NF-kappaB and active NF-kappaB complexes activation in the nucleus.

Theses studies and our results point a major IkappaB-β role in persistent NF-kappaB activation. The presence of the two IkappaB molecules should allow greater flexibility in the Rel transcription factor regulation, since both inhibitors respond differentially to proliferating stimuli [25]. These capacities of IkappaB's have been tested; IkappaB-α is a 10 times more efficient inhibitor of NF-kappaB than IkappaB-β, a capacity which results from the ability to remove NF-kappaB from DNA [26]. Possibly, IkappaB-α corresponds to stress situations, while the NF-kappaB persistent activation is an obliged response to the chronic inflammation condition, infection, differentiation and carcinogenesis. It has been shown that p65/IkappaB-β complexes are associated with kappaB-Ras proteins [27]. These ternary complexes respond differentially to extracellular signals, due to the incapacity of IKK to phosphorylate IkappaB-β in presence of k-Ras, sice kappaB-Ras masks the exposed p65 nuclear localization signal (NLS) [28]. It's important to point out that binary IkappaB-β/NF-kappaB complexes remain entirely in the cytosol, although IkappaB-α complexes wait around close to the nucleus, a difference which may confer more efficient activity [29]. In order to explain the different inhibition capacities of IkappaB, a novel computational program has been developed to predict temporal control to NF-kappaB activation based on coordinate degradation of both IkappaB. This model indicates a certain responsibility on the part of IkappaB-α for the negative feedback which leads to a transitory NF-kappaB activation, while IkappaB-β works to reduce the oscillatory potential of the system and to stabilize the NF-kappaB response during prolonged stimuli [30].

## Conclusion

In summary, our results have identified that NF-kappaB complexes responds in various ways signalling differences during early stages in the hepatocarcinogenesis model versus a sole liver regeneration induced by PH. In the hepatocarcinogenesis model, the IkappaB-α degradation turns out to be acute and transitory, and IkappaB-β degradation persists for long periods. After the sole PH, IkappaB-β is not degraded, only degradation of IkappaB-α is observed, which is very likely to stimulate transitorily transcription factors response, and to assure the blockade of NF-kappaB up until recovering the total mass of the liver. We suggest that during persistent NF-kappaB activation in the early stages of hepatocarcinogenesis, the participation of IkappaB-β degradation is important for contributing to the proliferation of transcription genes needed for neoplastic proliferation. These results open the possibility that IkappaB-β degradation is caused by alternative activation pathways other than IkappaB-α signalling in early hepatocarcinogenesis.

## Methods

### Animal treatment and experimental groups

Male Fisher rats 344 weighing 180–200 g were housed in a room at constant temperature. They had free access to water and laboratory diet food. Forty five animals were randomly assigned to 5 groups, it is to say, 3 rats in every time of sacrifice. The control group (CN) received no treatment (n = 3). The second group received one sole dose of diethylnitrosamine (DEN) (200 mg/kg) ip as inititator and was sacrificed 7 d after DEN administration (n = 3). The third group, was treated with one sole dose of DEN (200 mg/kg) ip and seven days after DEN administration, they received three daily oral serial doses of a suspension of 2-acetylaminofluorene (2-AAF) (SIGMA-ALDRICH Quimica) as promotion stimulus, at dose of 25 mg/kg and were sacrificed 24 h after the last dose of 2-AAF. The fourth group, the complete treatment group (TC), received treatment which induces liver cancer according to a modified version of the Solt and Farber model [10]. These animals were initiated using DEN (200 mg/kg) ip and on the seven day after DEN administration, they were promoted with an oral suspension of 2-AAF, at dose of 25 mg/kg for three consecutives days. On day 10 animals underwent two-thirds partial hepatectomy (PH) as proliferating stimulus and this group was sacrificed at different periods at 15, 30 and 60 min; 24, 48 h and 15 d after partial hepatectomy. The fifth group was treated with only PH. The animals of this group were sacrificed at different periods after PH, at 15, 30 and 60 min; 24, 48 h and 15 d. Livers were washed in physiological saline solution and frozen in liquid nitrogen and other sections were frozen in 2-methylbutane to crio-protection and stored at -79°C. The figure 1 shows the experimental design for each group, and the times of sacrifice are indicated with this symbol: . The 100% of the animals survived up to sacrifice time.

### Western-blot analysis

For the analysis of Rel A/p65, IkappaB-α and IkappaB-β, nuclear and cytosolic extracts were prepared from frozen liver samples [31,32]. Twenty μg of extracts proteins were separated using 10% SDS-PAGE for p65 and at 12% for IkappaB-α and β. The separated proteins were transferred to PVDF membranes. Non-specific binding was blocked by pre-incubation of the PVDF in 1% non-fat milk (5% w/v), Tween 20 (0.1% v/v) in phosphate buffered saline before being incubated overnight at 4°C with polyclonal anti-p65 (Upstate, NY, USA), anti-IkappaB-α and anti-IkappaB-β (Santa Cruz Biotechnology, Santa Cruz, CA) antibodies, in a 3% non-fat milk and 0.05% albumin bovine (Sigma-Aldrich) Tween 20 in phosphatase-buffered saline. Bound primary antibody was detected using horseradish peroxidase conjugated anti-rabbit and anti-mouse antibodies (Santa Cruz Biotechnology, Santa Cruz, CA) by chemiluminiscence using the Luminol kit (Santa Cruz Biotechnology, Santa Cruz, CA) and developed in a photographic plaque (Konica, JP). The samples were normalized using β-actin and lamin B (Santa Cruz Biotechnology, Santa Cruz, CA) for cytoplasm and nuclear extracts respectively. The density of the specific Rel A/p65 (65 kD), IkappaB-α (45 kD) and β (35 kD) bands, was quantified using an imaging densitometer software (analysis Imaging System, GmbH).

### Electrophoretic mobility shift assay (EMSA)

Nuclear extracts were incubated with consensus oligonucleotides of kappaB site (5'-AGC TAA GGG ACT TTC CGC TGG GGA CTT TCC AG-3') (Invitrogen Corp. N.Y.). The oligonucleotides were labeled with {γ-^32^P} by T_4 _polynucleotide kinase for 1 h at 37°C. Binding reactions included 10 μg of nuclear protein in incubation buffer (HEPES 120 mmol/L, EDTA 1 mmol/L, glycerol 25%, MgCl2 8 mmol/L, KCl 60 mmol/L and Tris-HCl 40 mmol/L), spermidine 10 mmol/L, poly dI:dC 0.5 μg (Invitrogen Corp. NY) and 50 ng of the oligonucleotide labelled kappaB site. The samples were electrophoresed through a 6% polyacrylamide gel for 2 h at 100 EV. The gel was dried in a vacuum and autoradiographed at -70°C for 12 h. The bands were densitometrically analyzed.

### Reverse transcription-polymerase chain reaction (RT-PCR)

The total RNA extraction and electrophoresis gel agarose were elaborated using standard methods [12,13]. The cDNA synthesis and PCR were performed in one step (One step RT-PCR System, Gibco Invitrigen Corp. NY). To the reaction mix were added 0.2 μmol/L of primer for *IkappaB-α *gene, and 0.04 μg of total RNA. To verify contamination with DNA, samples were analyzed using PCR assay. The program cycle consisted of 45 min at 45°C for cDNA synthesis, 2 min at 94°C followed by 45 s at 94°C for denaturalization, the annealing time was 45 s at 63°C, and 15 min at 72°C for final elongation. To find an adequate exponential amplification region to avoid the saturation of the system, a kinetic study of amplification related to the number of cycles was performed. The sequence primers were: forward 5'-CATGAAGAGAAGACACTGACCATGGAAA-3' and reverse 3'-TGGATAGAGGCTAAGTGT AGACACG-5', the product size was 329 pb.

### Statistical analysis

Mean and standard deviation were calculated and their variance evaluated by means of ANOVA test. A difference was considered significant when P value was less than 0.05. The statistical software used was STAT 32 (SPSS Inc.).

## Authors' contributions

JIPC participated in the design of the some experiments and organized the physiological data. AMQ was involved in RT-PCR experiments. In the inmmunoblots experiments was involved heavily MESN. SVT was involved heavily in the editing process and final draft production.
